# Quartz Crystal Microbalance as a Holistic Detector for Quantifying Complex Organic Matrices during Liquid Chromatography: 2. Compound-Specific Isotope Analysis

**DOI:** 10.1021/acs.analchem.3c05441

**Published:** 2024-05-03

**Authors:** Christopher Wabnitz, Wei Chen, Martin Elsner, Rani Bakkour

**Affiliations:** Department of Chemistry, Chair of Analytical Chemistry and Water Chemistry, TUM School of Natural Sciences, Technical University of Munich, Lichtenbergstr. 4, 85748 Garching, Germany

## Abstract

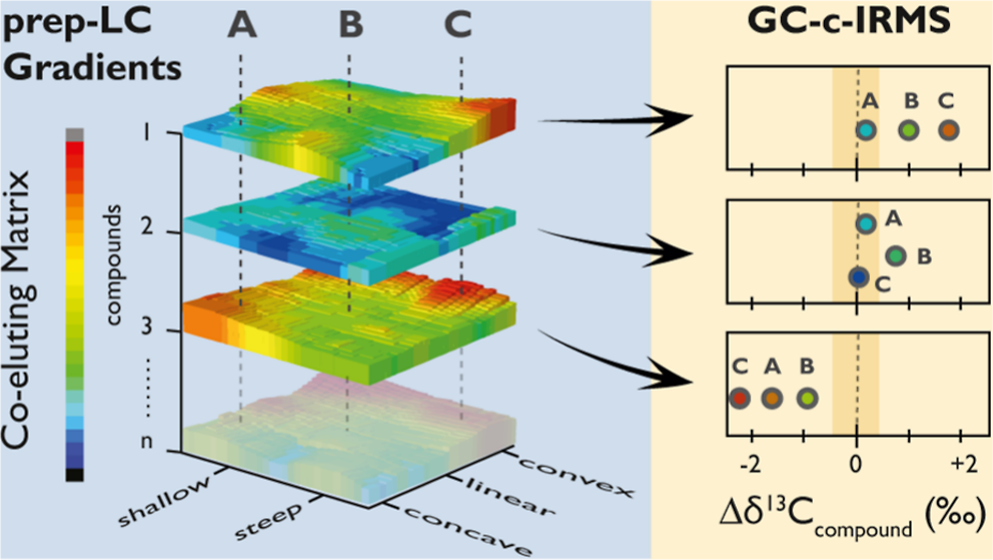

In carbon-compound-specific isotope analysis (carbon CSIA) of environmental micropollutants, purification of samples is often required to guarantee accurate measurements of a target compound. A companion paper has brought forward an innovative approach to couple a quartz crystal microbalance (QCM) with high-performance liquid chromatography (HPLC) for the online quantification of matrices during a gradient HPLC purification. This work investigates the benefit for isotope analysis of polar micropollutants typically present in environmental samples. Here, we studied the impact of the natural organic matter (NOM) on the isotopic integrity of model analytes and the suitability of the NOM-to-analyte ratio as a proxy for the sample purity. We further investigated limitations and enhancement of HPLC purification using QCM on C_18_ and C_8_ phases for single and multiple targets. Strong isotopic shifts of up to 3.3% toward the isotopic signature of NOM were observed for samples with an NOM-to-analyte ratio ≥10. Thanks to QCM, optimization of matrix removal of up to 99.8% of NOM was possible for late-eluting compounds. The efficiency of HPLC purification deteriorated when aiming for simultaneous purification of two or three compounds, leading to up to 2.5% less NOM removal. Our results suggest that one optimized HPLC purification can be achieved through systematic screening of 3 to 5 different gradients, thereby leading to a shift of the boundaries of accurate carbon CSIA by up to 2 orders of magnitude toward lower micropollutant concentrations.

## Introduction

Compound-specific isotope analysis (CSIA) has proven to be a powerful tool for identifying environmental contamination sources and delineating their natural and engineered degradation pathways by measuring isotopic ratios (i.e., ^13^C/^12^C, ^15^N/^14^N) of the contaminant/target analyte at natural abundance.^1−11^ To this end, gas chromatography combustion isotope ratio mass spectrometry (GC-c-IRMS) is typically used, where the contaminant is separated from other components in the sample using a gas chromatograph, then converted in a combustion oven to a universal gas (i.e., CO_2_, N_2_), and measured using a sector-field mass spectrometer.^12−15^ The structural information on the compound gets, however, lost during this process, which makes accurate CSIA susceptible to interferences by concurrent carbon-/nitrogen-containing constituents in the same sample.^1,16,17^ This represents a challenge for carbon CSIA of polar environmental contaminants that occur in low concentrations, such as pesticides and pharmaceuticals, for the following reasons. (i) The concentrations of such contaminants occur in the ng/L to μg/L range, whereas the potential interferences, namely, natural organic matter (NOM), occur at 10^3^ to 10^6^ higher concentrations in the mg/L range.^18^ (ii) The heterogeneity of NOM, which consists of thousands of different organic compounds found in environmental samples like river water, renders an efficient separation of the target analyte and interferences challenging in one extraction step.^19,20^ While classical mass spectrometry can correct for adverse effects caused by such interferences using, for example, internal standards,^21−24^ analyte protectants,^25,26^ or matrix-matched calibration,^21,24,27^ such corrections are not possible in GC-c-IRMS. Therefore, CSIA critically depends on highly purified samples.

Several purification strategies are at the analyst’s disposal to separate target analytes from sample interferences, also referred to as a matrix, in carbon-CSIA sample preparation. These strategies range from offline chromatographic techniques using conventional solid-phase extraction (SPE) materials,^28,29^ molecularly imprinted polymers (MIPs),^30^ cyclodextrin polymers,^20^ immunoaffinity chromatography,^31^ silica gel chromatography,^32,33^ or ion-exchange chromatography^34^ to different types of online chromatographic purification techniques including size-exclusion chromatography^35^ or the most widely used reversed-phase (RP) high-performance liquid chromatography (HPLC).^29,36−42^ While the target analytes are monitored in most of these works, this is not necessarily the case for all interferences. For example, consider ^13^C/^12^C measurement of atrazine in a groundwater extract containing interfering NOM, where chromatographic cleanup is warranted prior to GC-c-IRMS. To screen for the optimal cleanup conditions, fractions have to be collected, organic solvents removed, the sample reconstituted in water, and each fraction measured using a total organic carbon (TOC) analyzer. On the other hand, quantification of matrix interferences during online purification procedures like HPLC is often hindered by a lack of suitable detectors.^43^ Detectors usually combined with HPLC are only able to monitor specific fractions of common matrices like NOM (i.e., chromophoric, fluorescent, or ionizable)^44,45^ or show intercompound response differences, as discussed in detail in the companion paper,^43^ among others.^45−48^ Alternatively, monitoring and quantifying potential interferences during the purification would, in fact, give insights into the success of the cleanup and its exact gain. We have brought forward in the companion study an innovative approach using quartz crystal microbalance (QCM) dry mass sensing that was coupled to RP HPLC and used to monitor and gravimetrically quantify NOM during a gradient HPLC purification.^43^

In the current and the companion study,^43^ we spray a small part (<1%) of the column effluent on a QCM using a microfluidic spray-dryer, adopting elements of other works by Schulz and King,^49^ Müller et al.,^50^ and Kartanas et al.^51^ In this process, the column effluent is nebulized into micron-sized droplets, which leads to the immediate evaporation of the solvent and deposits the nonvolatile components on the QCM. Their absolute mass is measured due to the QCM’s ability to measure mass changes on the oscillating piezoelectric quartz crystal with subnanogram resolution.^52,53^ Using this approach, it was possible to quantify matrix interferences in real time during RP HPLC. This approach, hence, circumvented challenges for QCM dry mass sensing before application to RP HPLC cleanup through (i) enabling the use of organic solvents including gradients by using a microfluidic spray-dryer, (ii) characterizing variations of the QCM response caused by gradients, and (iii) alleviating the impact of the latter through a suitable calibration strategy.^43^ It seems ideal to apply this approach for environmental extracts intended for carbon-CSIA measurements after RP HPLC. Yet, a quantitative assessment of the gain of optimizing RP HPLC purification using real-time matrix monitoring has never been conducted—and its impact on accurate carbon CSIA has never been explored.

The work presented in this and the companion paper^43^ has the overall goal of exploring the feasibility of coupling a commercial high-performance liquid chromatograph with a microfluidic spray-dryer and a QCM for online monitoring of organic matrix components during RP HPLC gradient purification for mass spectrometry-based applications in environmental sciences. Both studies focus on organic matrices in already extracted samples, where most inorganic salts are excluded through a first SPE step. While the technical and fundamental groundwork for matrix online monitoring using QCM dry mass sensing during RP HPLC was laid out in the companion study,^43^ this paper systematically investigates the purification potential of RP HPLC before ^13^C/^12^C analysis of polar micropollutants present in environmental water samples using GC-c-IRMS. To this end, we (i) studied the impact of NOM on the isotopic integrity of model analytes and whether the NOM-to-analyte ratio (*C*_NOM_/*C*_analyte_, nmol C/nmol C) can be used as a proxy for the sample purity and (ii) investigated limitations and enhancement of HPLC purification using QCM dry mass sensing on C_18_ and C_8_ phases for single and multiple targets.

## Experimental Section

### Chemicals, Materials, and Samples

A list of purchased chemicals and materials, a description of standard solutions, and working solutions used in this study are provided in the Supporting Information (Section S1). NOM was extracted from surface water samples as detailed in the companion paper^43^ and summarized in Section S2. Samples for isotope analysis with different NOM/analyte ratios (10, 20, 50, and 100), *C*_NOM_/*C*_analyte_ in mol C/mol C, were prepared in methanol by mixing the stock solution of extracted NOM with stock solution of the corresponding analyte to reach an analyte concentration of 1667 nmol C/mL, corresponding to 5 nmol C per injection on GC-c-IRMS. Extracts for HPLC purification were prepared in methanol/water (25/75 v/v) by spiking extracts of river water containing 9000 mg/L NOM with eight different model analytes, namely, 2,6-dichlorobenzamide (BAM), atrazine (ATZ), azoxystrobin (AZOX), boscalid (BOSC), caffeine (CAF), desethylatrazine (DEA), desisopropylatrazine (DIA), and simazine (SIM), 3 mg/L each. These extracts correspond to original water samples with 3.6 mg/L NOM and 120 ng/L analyte.

### Chemical Analysis

#### Compound-Specific Isotope Analysis

Carbon isotope measurements were performed on a GC-c-IRMS system consisting of a gas chromatograph (TRACE GC Ultra multichannel gas chromatograph, Thermo Fisher Scientific, Germany; Column: J&W DB-5MS UI column, *L* = 30 m × ID = 0.25 × film thickness = 1.0 μm, Agilent, Germany), a combustion interface (see details in section S3, Finnigan GC Combustion III Interface, Thermo Fisher Scientific, Germany), and an isotope ratio mass spectrometer (Finnigan MAT 253 IRMS, Thermo Fisher Scientific, Germany). Extracts in methanol were injected (3 μL injection volume) using an autosampler (GC PAL, CTC, Switzerland) with splitless injection mode (liner: ID = 5 mm × *L* = 105 mm, Thermo Fisher Scientific, Germany) at 250 °C and a surge pressure of 250 kPa. Analytes were separated at a helium flow of 1.4 mL/min using the temperature program detailed in Section S3. The peaks were automatically detected and baseline corrected (individual background algorithm) using the Isodat software of Thermo Fisher Scientific, Germany. Isotope ratios were calculated in relation to a CO_2_ reference gas (Carbo, Germany) and are reported as arithmetic means of at least triplicate measurements as δ ^13^C values (in ‰) with the respective 95% confidence interval (CI) relative to the international reference material Vienna PeeDee Belemnite (VPDB).^54^ In addition, standard bracketing procedures were used to ensure identical treatment of the standard and sample^55^ and method quantification limits were determined according to the moving mean procedure (see Figure S1 and Table S5).^56^

#### High-Performance Liquid Chromatography

A Nexera XR HPLC system (Shimadzu, Japan) was used for chromatographic separation. It consists of a solvent delivery module (LC-20AD, Shimadzu, Japan), a diode array detector (DAD) (SPD-M20A, Shimadzu, Japan), and a fraction collector (FRC-10A, Shimadzu, Japan). As the stationary phase, two different columns were used: XTerra RP18 column (particle size = 3.5 μm, *L* × *D* = 150 × 3.0 mm, pore size = 125 Å, Waters, USA) and Orbit 100 C8 column (particle size = 3.5 μm, *L* × *D* = 150 × 3.0 mm, pore size = 100 Å, MZ Analysentechnik, Germany). As the mobile phase, binary gradients consisting of water (A) and methanol/water (90/10 v/v) (B) were used. A column oven temperature of 40 °C, a flow rate of 0.5 mL/min, and a sample injection volume of 200 μL were used for all measurements. Using the DAD, the retention time and peak width of each analyte were determined at the corresponding maximum absorption wavelength and used to constrain the fraction in which the analyte was completely recovered. For HPLC optimization, the RP gradient conditions were systematically varied by changing the percentage of CH_3_OH in the mobile phase at minute 7.5 (30, 40, 50, 60, 70, 80, or 90%) and minute 15 (60, 70, 80, or 90%) covering, thereby, linear, concave, and convex gradients. Twenty-two and 7 different gradients were studied for the XTerra RP18 column and Orbit 100 C8 column, respectively (see Tables S7 and S8).

#### QCM Dry Mass Sensing Coupled to HPLC

The QCM dry mass sensing system was coupled to the HPLC system, characterized, and validated as described in detail in the companion paper.^43^ In short, the HPLC effluent was split after the DAD and prior to the fraction collector using a postcolumn adjustable flow splitter. The high-flow port was connected to the fraction collector, whereas the low-flow port was connected to a microfluidic spray-dryer. The latter was fabricated in-house using a standard polydimethylsiloxane (PDMS) soft lithography approach.^43^ Using the spray-dryer, the HPLC effluent was sprayed onto a 5 MHz QCM crystal (100RX1, Cr/Au, Stanford Research Systems, USA) placed in a frequency counter (QCM200, gate time: 0.1 s, Stanford Research Systems, USA). Each measurement consisted of a blank run (methanol/water 25/75 v/v), the sample (NOM-containing extract), and a one-point calibration (c(NaCl) = 300 mg/L in the mobile phase), which were used to derive the concentration of the matrix in milligrams per liter in the sample during chromatography.

### Data Evaluation

#### QCM Dry Mass Sensing

The QCM dry mass sensing data was evaluated using a MATLAB script as reported in the companion paper.^43^ In short, after correcting the frequency measurement of the sample and that of the calibration using one of the blank, the first derivative was derived from the corrected frequencies. Then, the first derivatives were smoothed using a Savitzky–Golay filter. To get the mass concentration of the sample in mg/L, the smoothed first derivative of the sample measurement was divided by the smoothed first derivative of the calibration measurement and multiplied by the concentration of the calibration solution (see eq 1 in the companion paper^43^).

#### *C*_NOM_/*C*_analyte_ Ratio and the Gain Factor

The *C*_NOM_/*C*_analyte_ ratio in mol C/mol C before HPLC purification ([*C*_NOM_/*C*_analyte_]_no LC_) was calculated by dividing the molar concentration of NOM by the molar concentration of the respective analyte in the extract. To calculate the *C*_NOM_/*C*_analyte_ ratio after HPLC purification ([*C*_NOM_/*C*_analyte_]_LC_), the integral of the NOM data measured using QCM dry mass sensing during HPLC purification was taken over the corresponding time window of the analyte peak (area_fraction_). The latter was divided by the integral of the NOM data over the whole chromatogram (area_total_), where complete recovery of NOM was validated, to get the percentage of NOM coeluting during the analyte fraction (see eq 1)
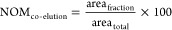
1

[*C*_NOM_/*C*_analyte_]_no LC_ was multiplied by the percentage of NOM coeluting in the respective fraction to get [*C*_NOM_/*C*_analyte_]_LC_ (see eq 2)
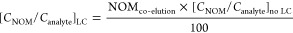
2

The gain factor, which is the factor by which *C*_NOM_/*C*_analyte_ was improved, was calculated by dividing [*C*_NOM_/*C*_analyte_]_no LC_ by [*C*_NOM_/*C*_analyte_]_LC_ (see eq 3)
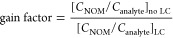
3

#### Matrix Removal for Individual and Multiple Compounds

The matrix removal in % for individual compounds was calculated by subtracting the percentage of coeluting NOM from 100 (see eq 4)
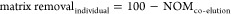
4

To determine the maximal matrix removal during multiple compound purification, we added for each investigated HPLC gradient the respective matrix removal of the individual compounds and divided the value by the number of compounds *n* to get the average matrix removal for n-compounds (matrix removal_*n*-compounds_) (see eq 5; see examples in Figures S16 and S17)
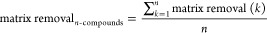
5

This calculation was made for each HPLC gradient separately. The gradient with the highest matrix removal of compounds_*n*-compounds_ (gradient_*m*_) was selected as the optimal gradient for the respective combination of compounds. The exact matrix removal of each of the compounds for gradient_*m*_ was used as the maximal matrix removal for this purification problem. The difference between the optimal gradient determined for the individual compound (gradient_*i*_) and gradient_*m*_ is reported as a loss in the matrix removal. Repeating this procedure for several combinations of two or three early-, middle-, and late-eluting compounds (Tables S17 and S18) made it possible to determine an average matrix removal and to plot the different determined numbers in a box plot (see Figure 3).

## Results and Discussion

### Natural Organic Matter-to-Analyte Ratio as Proxy for Sample Purity and Its Impact on Isotopic Integrity

**Figure 1:**
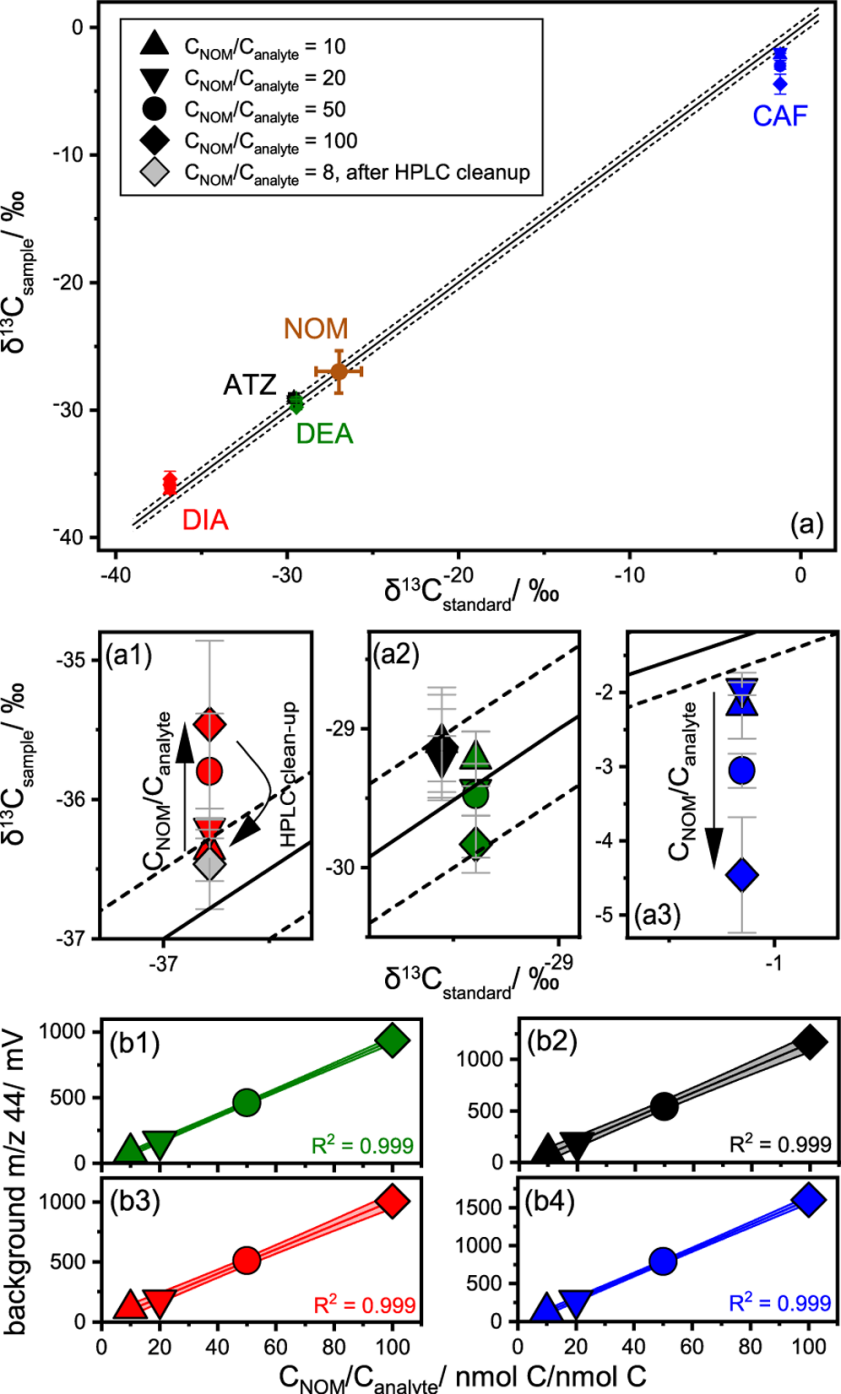
(a) The isotope value of standard measurements of four different analytes (DEA: green, ATZ: black, DIA: red, CAF: blue) is plotted against the isotope value measured in extracts containing NOM in different *C*_NOM_/*C*_analyte_ ratios (10: triangle up, 20: triangle down, 50: circle, 100: diamond). The range of typical NOM isotope values (δ ^13^C = 27 ± 1‰) is highlighted (brown circle). (a1–a3) Enlarged areas of the four analytes. (a1) Gray: Extract with concentration of NOM equal to ratio 100 was subjected to HPLC cleanup using XTerra RP18 (see the HPLC gradient in Table S4). The respective fraction of DIA was collected, the solvents were evaporated, and NOM was reconstituted and spiked with DIA to reach an analyte concentration of 1667 nmol C/mL and a total volume equal to the original NOM extract (200 μL). (b1–b4) Correlation of the background intensity (*m*/*z* 44/mV) at the respective analyte retention time in the GC-c-IRMS chromatogram and the amount of NOM injected.

We assessed the NOM/analyte ratio, *C*_NOM_/*C*_analyte_ in mol C/mol C, as a representative indicator of sample purity and its impact on accurate isotope analysis. Figure 1a shows measured δ ^13^C values on GC-c-IRMS of four different model analytes, namely, DIA (δ ^13^C = −36.8 ± 0.5‰), ATZ (−29.6 ± 0.5‰), DEA (−29.4 ± 0.5‰), and CAF (δ ^13^C = −1.2 ± 0.5‰), in extracts containing different *C*_NOM_/*C*_analyte_ ratios (10, 20, 50, and 100 mol C/mol C) and compared to standard measurements in the absence of NOM. Analyte concentrations were kept constant for all samples at 5 nmol C injected in each measurement, corresponding to concentrations of 57.9 mg/L (DIA), 44.9 mg/L (ATZ), 52.1 mg/L (DEA), and 40.5 mg/L (CAF) in the extract. The corresponding background intensities at *m*/*z* 44 are depicted in Figure 1b at the respective analyte retention time in the GC-c-IRMS chromatogram as a function of *C*_NOM_/*C*_analyte_.

We observed significant δ ^13^C shifts in the presence of NOM for DIA (Figure 1a1, red data, positive shift) and CAF (Figure 1a3, blue data, negative shift), while no significant shifts are visible for ATZ and DEA (Figure 1a2, black and green data). The absence of isotopic shifts for ATZ and DEA confirms the observation of Glöckler et al.,^20^ where compounds with an isotopic signature close to the one of NOM do not suffer from isotopic shifts induced by the sample matrix. Indeed, δ ^13^C of ATZ (−29.6 ± 0.5‰) and DEA (−29.4 ± 0.5‰) are both in the proximity of that of NOM (−27 ± 1‰)^57^ on the carbon isotopic scale. This implies that the obtained δ ^13^C values of the analytes are not only attributable to the compound but also, strictly speaking, to a bulk measurement of the analyte and matrix. In contrast, the effect of NOM on δ ^13^C integrity of DIA (−36.8 ± 0.5‰) and CAF (−1.2 ± 0.5‰) is evident and becomes most pronounced when the distance between the isotopic signature of the target analyte and that of NOM is further apart. This is corroborated by the direction of the isotopic shift, which consistently goes in the direction of the isotopic signature of NOM (positive for DIA, negative for CAF), and the magnitude of the shift, which is greater for CAF (δ ^13^C_sample_ – δ ^13^C_standard_ for *C*_NOM_/*C*_analyte_ ratio 100: −3.3 ± 0.8‰) compared to DIA (+1.3 ± 0.6‰) reflecting the greater difference to the one of NOM on the isotopic scale (CAF-NOM = +25.8 ± 1.1, DIA-NOM = −9.8 ± 1.1). Even the magnitude of isotopic shifts is progressively following the *C*_NOM_/*C*_analyte_ ratios (see the arrow in Figures 1a1,a3 and S2 in the Supporting Information). The observed deviation is, however, not precisely the composite of the background and peak due to the applied individual background algorithm implemented in the Isodat software.

The influence of the matrix NOM on the measurement can also be seen in the IRMS chromatograms. A distinct hump-shaped baseline rise is visible in the samples containing NOM (Figure S3). We found a direct correlation (*R*^2^ ≥ 0.999) between the amount of NOM injected and the background intensity (*m*/*z* 44) recorded on the IRMS at the respective analyte retention times for all compounds (see Figure 1b). Consequently, the ratio of the injected matrix and analyte, *C*_NOM_/*C*_analyte_, seems to be a good proxy of the sample purity as proposed by Bakkour et al.^30^ and Glöckler et al.^20^ Accurate isotope values of DIA were only measured for *C*_NOM_/*C*_analyte_ ≤ 10 (Figures 1a1 and S2). To probe further, we moved the *C*_NOM_/*C*_analyte_ ratio from 100 to 8 using HPLC purification and were thus able to recover the isotope integrity of the analyte (Figure 1a1 gray data point). For CAF, a *C*_NOM_/*C*_analyte_ of 10 was not sufficient to resolve the target analyte peak and guarantee accurate isotope analysis (Figures 1a3 and S2). The exact *C*_NOM_/*C*_analyte_ ratio guaranteeing accurate isotope analysis varies depending on the analyte, the distance between the signature of the analyte and NOM on the isotopic scale, and the GC method. This highlights the importance of (i) including standards spanning over a range of isotope signatures in carbon-CSIA method development and (ii) the purity of the sample as a strategy to avoid systematic bias in isotope values.

### Limitations and Enhancement of Preparative Chromatography Revealed by QCM Dry Mass Sensing

**Figure 2:**
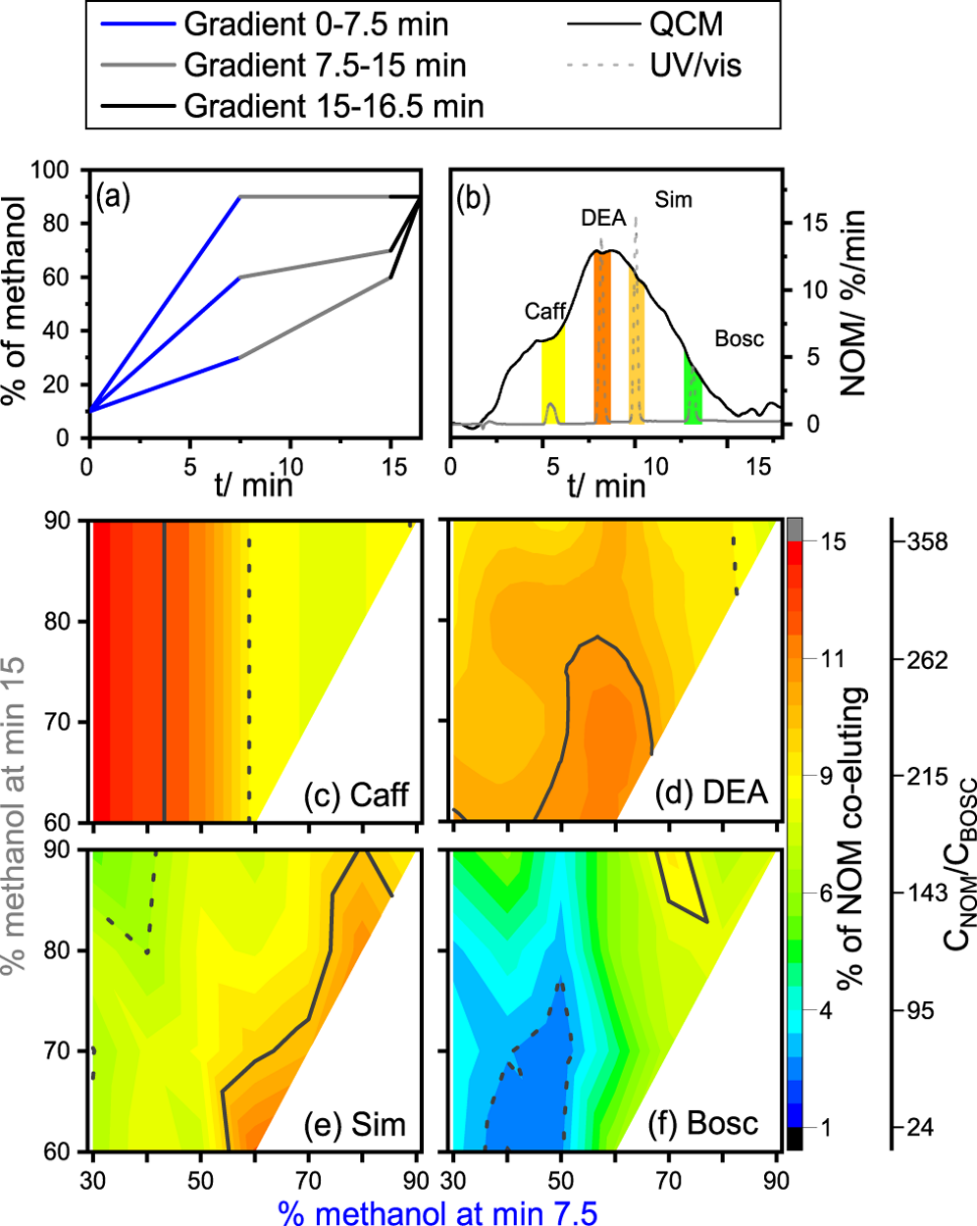
(a) Three out of the 22 measured gradients with varying % of CH_3_OH in the mobile phase until minute 7.5 (blue; 30, 40, 50, 60, 70, 80, or 90%) and minute 15 (gray; 60, 70, 80, or 90%). (b) Exemplary chromatogram (gradient 10–60–70) shows the analyte peaks constrained using UV/vis for CAF, DEA, SIM, and BOSC (dotted gray line) and NOM in %/min quantified using QCM dry mass sensing (black line). The amount of coeluting NOM during the analyte retention window is integrated (colored areas) and divided by the total amount of NOM measured to receive a number of the percentage of NOM coeluting with the analyte (corresponding color). (c–f) The NOM coelution in % is plotted for the 22 different gradients for 4 analytes [(c): CAF, (d): DEA, (e): SIM, (f): BOSC]. The second axis shows the *C*_NOM_/*C*_analyte_ ratio after the purification step corresponding to BOSC ratio in the original extract of 2383. The minima are encircled using a black dotted line, and the maxima are encircled using a black solid line.

To quantitatively assess the limits and possible enhancement of typical preparative chromatography cleanup steps in removing the organic matrix from a sample extract, we selected 8 model compounds (CAF, BAM, DIA, DEA, SIM, ATZ, AZOX, BOSC; log *K*_OW_ range: −0.07 to 2.96) spiked to an extract containing NOM as an organic matrix and subjected them to HPLC cleanup using a C_18_ stationary phase (XTerra RP18), a classical phase used in many CSIA applications (see Table S19). Binary solvent mixtures of water and CH_3_OH were systematically varied by changing the percentage of CH_3_OH in the mobile phase at minute 7.5 and minute 15, thus covering linear, concave, and convex gradients (see illustrative gradients in Figure 2a; all gradients in Tables S7 and S8). NOM concentrations in the HPLC effluent were acquired using QCM dry mass sensing, whereas analyte retention times were monitored using UV–visible spectroscopy (UV/vis) detection at the corresponding maximum absorption wavelength.

For illustrative purposes, we reduced the complexity of Figure 2 by showing data only for CAF, DEA, SIM, and BOSC, whereas the data for remaining analytes are shown in Figure S4 with a detailed summary in Tables S10 and S11. Considering the example of CAF, data acquired from gradient 10–60–70 (Figure 2a, middle gradient) are shown for the four analytes in Figure 2b where the CAF fraction is completely collected around min 5 (Figure 2b, yellow region). According to QCM-acquired data (Figure 2c, *x* = 60%, *y* = 70%), this specific fraction contains around 8% of the originally injected NOM (heat map scale).

#### Gains from an Individual Compound Perspective

A single HPLC purification of an extract of a 5 L water sample containing 1.8 mgC/L NOM (postspiked after the extraction with each respective analyte to correspond to 120 ng/L in the original water sample) could remove between 85 and 91% of the coextracted NOM (see Table 1, “LC_XTerra RP18_ matrix removal”). This corresponds to a remaining percentage of coeluting NOM in each fraction of between 9 and 15% of the original NOM concentration (see Figure 2c–f solid marked areas). The *C*_NOM_/*C*_analyte_ ratio in the extract could thus be reduced by a factor of 7 to 12 from ratios ranging between 2292 and 4343 to a range between 207 and 548.

**Table 1:** Reduction of the *C*_NOM_/*C*_analyte_ Ratio during HPLC Purification of the Oasis HLB Extract of a Water Sample Containing 120 ng/L of Each Respective Analyte and 1.8 mgC/L NOM^a^

	early eluting			middle eluting		late eluting		
	CAF	BAM	DIA	DEA	SIM	ATZ	AZOX	BOSC
log *K*_ow_	–0.07	0.77	1.50	1.51	2.18	2.61	2.50	2.96
no LC	3034	3393	4343	3909	3601	3370	2292	2383
								
LC_XTerra RP18_	448	347	548	453	417	403	264	207
gain factor	7	10	8	9	9	8	9	12
matrix removal (%)	(85.3)	(89.8)	(87.4)	(88.4)	(88.4)	(88.0)	(88.5)	(91.3)
								
optim-LC_XTerra RP18_	242	215	296	281	192	107	53	47
gain factor	13	16	15	14	19	31	44	51
matrix removal (%)	(92.0)	(93.7)	(93.2)	(92.8)	(94.7)	(96.8)	(97.7)	(98.0)
								
optim-LC_Orbit 100 C8_	96	156	206	271	129	78	14	**4**
gain factor	32	22	21	14	28	44	167	556
matrix removal (%)	(96.8)	(95.4)	(95.3)	(93.1)	(96.4)	(97.7)	(99.4)	(**99.8**)

a The table displays the *C*_NOM_/*C*_analyte_ (nmol C/nmol C) ratio in the extract (no LC), the reduced ratio for the gradient on XTerra RP18 that showed the highest (LC_XTerra RP18_) and the lowest (optim-LC_XTerra RP18_) NOM coextraction and the lowest (optim-LC_Orbit 100 C8_) coextraction on Orbit 100 C8. It also shows the gain factor, which is calculated by dividing the *C*_NOM_/*C*_analyte_ ratio before the cleanup (“no LC”) by the *C*_NOM_/*C*_analyte_ ratio after the respective cleanup. The matrix removal in % is shown in brackets. The analytes are classified in early-, middle-, and late-eluting substances depending on their retention behavior during the 22 investigated gradients and listed in the order of their retention time.

While these results show the substantial purification potential of HPLC using a typical C_18_ column without any method development, the *C*_NOM_/*C*_analyte_ ratio is still too high for accurate carbon CSIA (≤10). This highlights the need for optimizing HPLC purification. In fact, screening for 22 gradients using QCM dry mass sensing led to an additional 6.7% NOM removal in the retention window of CAF, 3.7% in the window of DEA, 6.3% of SIM, and 6.7% of BOSC (see Figure 2c–f, dashed marked areas). These gains are significant considering the associated uncertainties between 0.1 and 1.1% according to triplicate to sextuplicate measurements (see Tables S10 and S11). Using the QCM-optimized HPLC purification, the C_NOM_/C_analyte_ ratios could be reduced to between 47 and 296 (“optim-LC_XTerra RP18_”), corresponding to gain factors between 13 and 51 compared to no cleanup (“no LC”) and to gain factors between 2 and 5 compared to not optimized LC (“LC_XTerra RP18_”).

An optimized single cleanup on XTerra RP18 leads to larger gain factors for late-eluting compounds (31–51), compared with early-eluting (13–16) and middle-eluting compounds (14–19). These results are meaningful given the shape of the NOM hump that can be influenced more for the late-eluting compounds than for the early and middle ones eluting directly with the main part of the NOM hump (see Figure 2b). Yet, *C*_NOM_/*C*_analyte_ = 47–296 is significantly above the required value for accurate carbon CSIA (≤10). This is not surprising given the concentration of the target analytes and NOM in the investigated water sample (1.8 mgC/L of NOM, 120 ng/L of analyte). This highlights that residuals of NOM as low as 2% in the collected fraction of such a sample require further optimization even when recovering 100% of the target analyte. Therefore, we assessed the potential for NOM removal on a different stationary HPLC phase, namely, an Orbit 100 C8 column, which offers a higher theoretical plate number (see Table S9) for the investigated compounds and, thereby, possesses a higher retention and smaller peak width presumably leading to an even lower NOM coelution and thus lower *C*_NOM_/*C*_analyte_ ratios. Indeed, it was possible to reach NOM removal of between 93.1 and 99.8% (see results for all gradients in S12 and S13) leading to gain factors for early- (21–32), middle- (14–28), and late-eluting compounds (167–556), as shown in Table 1 “optim-LC_Orbit 100 C8_”. Thanks to the QCM optimization, it was therefore possible to remove up to 99.8% of the matrix for BOSC with a single optimized cleanup leading to a *C*_NOM_/*C*_analyte_ ratio = 4, which is smaller than the suggested value of 10.

#### Trade-Offs between Single and Multiple Targets

**Figure 3:**
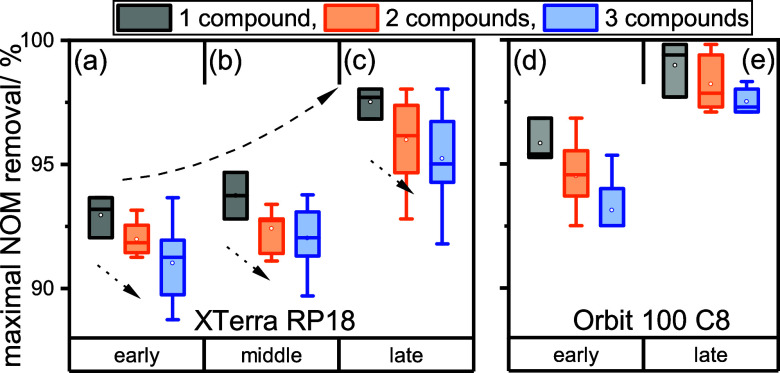
Removal of NOM (in %) in the fraction of early-, middle-, or late-eluting compounds during the purification of one individual compound (1: black) or multiple compounds (2: orange, 3: blue) for both columns. The dashed upward arrow annotates the trend of elution regions early < middle < late, and the dotted downward arrow annotates the compound number trend 1 > 2 > 3.

Purifying more than one compound in a single HPLC purification run is expected to lead to trade-offs in the potential of maximal NOM removal since (i) the optimal HPLC conditions identified for individual compounds (data shown in Table 1) do not necessarily coincide together (see Figures S16 and S17) and (ii) small variations in NOM coelution, as small as 1–2%, can be detrimental to accurate δ^13^C of the analyte as shown in the previous section. Therefore, we quantified the maximal NOM removal when optimizing HPLC purification for only one compound at a time and compared it with the NOM removal determined for the optimized purification for multiple targets over the whole chromatographic run, covering thereby combinations of early-, middle-, and late-eluting compounds (see Figure 3).

Efficiency of HPLC purification deteriorates when aiming for simultaneous purification of two (orange) or three compounds (blue) compared to an individual compound (black), as seen by the maximal NOM removal denoted as dotted downward arrows in Figure 3. For (a) early eluting compounds on XTerra RP18, 92.0% NOM can be removed on an average when purifying two compounds and 91.0% during the purification of three compared with one (93.0%). The same holds true for (b) middle- (1:93.7%, 2:92.4%, 3:92.0%) and (c) late-eluting compounds (1:97.5%, 2:96.0%, 3:95.2%). The trend of the NOM removal for individual and multiple compounds is consistent within each elution region following the order early < middle < late (denoted as dashed upward arrows). This picture may vary depending on the chromatographic behavior of different matrices, as well as on the exact combination of compounds used (see Tables S17 and S18 and Figures S14–S17). For example, the maximal NOM removal determined for late-eluting compounds in combination with middle-eluting ones (96.7%) is higher in comparison to the simultaneous purification with early-eluting compounds (95.3%).

Similar trends were observed on a different column, namely, Orbit 100 C8, which further corroborates the acquired results (see Figure 3d,e). The data on the middle-eluting compounds on Orbit 100 C8 is not shown since we did not determine any variations between the different combinations. Nonetheless, the determined maximal NOM removal on Orbit 100 C8 is higher in comparison with the XTerra RP18 column. In fact, the average NOM removal for three compounds on Orbit 100 C8 (“early”: 93.1%, “late”: 97.5%, see Figure 3d,e) is equal to the individual compound NOM removal on XTerra RP18 (“early”: 93.0%, “late”: 97.5%, see Figure 3a,c), highlighting the importance of the column choice. Although the differences in the maximal NOM removal for one, two, or three compounds might seem small, they are significant considering the precision of these measurements (±0.1–1.1%) and their impact on the *C*_NOM_/*C*_analyte_ ratios. This can be illustrated using the example of BOSC, where the *C*_NOM_/*C*_analyte_ ratio changes from 4 for the individual compound to 26 on an average for two compounds and to 44 on an average for three compounds, thus preventing accurate carbon-CSIA measurements in the latter cases.

## Conclusions and Analytical Implications

The present work systematically demonstrates that QCM dry mass sensing is a valuable auxiliary tool for optimizing matrix removal during a classical cleanup of extracts prior to carbon CSIA. In fact, this is the first study to report quantitative efficiencies of RP HPLC cleanup that amounted to matrix removal up to 99.8% upon optimization. On average, the maximal matrix removal within a precision of 1% could be determined by screening 3 to 5 different gradients, including convex, concave, and linear gradients (see Tables S14 and S15 and Figures S6–S13), thus demonstrating that a systematic method development with the help of QCM dry mass sensing yields substantial benefits with reasonable efforts.

**Figure 4:**
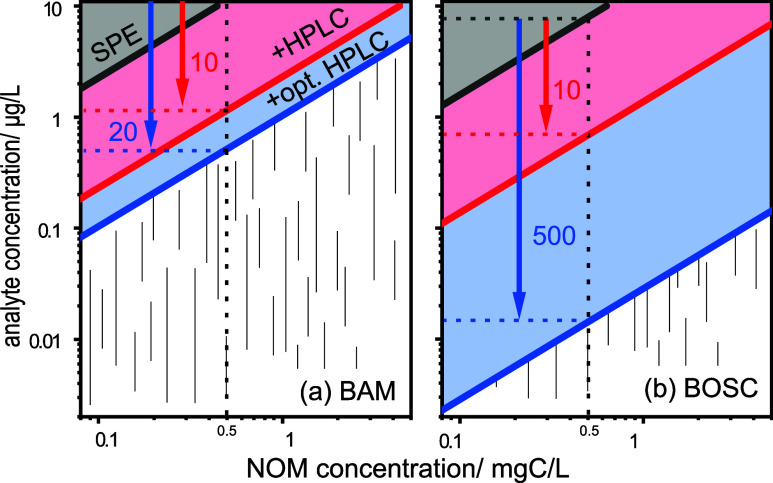
Dependence of accurate isotope analysis on the analyte and NOM concentration in the real-world water sample for (a) BAM (early eluting) and (b) BOSC (late eluting) for different sample preparation strategies: SPE using Oasis HLB (black), plus an HPLC purification (red), or plus an optimized HPLC purification (blue).

The discrepancies in gain factors of an HPLC cleanup between early- and late-eluting compounds have analytical implications for carbon CSIA. This is depicted in Figure 4 for one early-eluting compound (BAM, Figure 4a) and one late-eluting compound (BOSC, Figure 4b), where limits of accurate carbon CSIA are shown as a function of environmental analyte concentration (*x*-axis), NOM concentration (*y*-axis), and efficiency of the HPLC purification (red and blue arrows). While for both model compounds these limits can be shifted by approximately a factor of 10 to lower analyte concentrations using one HPLC purification (red arrow), a factor of up to 500 can be gained instead for a late-eluting compound by optimizing the HPLC purification (blue arrow). In contrast, only a factor of approximately 20 can be gained for an early-eluting compound. These findings are meaningful since the challenge of separating small polar compounds using RP columns is well known.^58−60^ Potentially, a column phase engineered for these compounds (e.g., HILIC)^61,62^ could result in a better separation of early-eluting compounds and NOM and thus a higher NOM removal during purification. To put these findings in a larger context of complete sample preparation for carbon CSIA, an overall higher removal can become possible when combining the targeted HPLC cleanup presented here with the use of more selective SPE materials (e.g., cyclodextrins)^20^ to replace Oasis HLB in the first extraction step, making it possible to measure concentrations ≥100 ng/L for BAM and ≥3 ng/L for BOSC in a groundwater sample containing 0.5 mgC/L NOM.

The use of NOM elution data for a given matrix is, furthermore, not limited to the 8 model compounds investigated in this study. Combined with software tools that can predict the analyte retention time and peak width,^63−65^ it is possible to determine the *C*_NOM_/*C*_analyte_ ratio for any given analyte and thus the feasibility of carbon CSIA. Creating in the future an openly available database for different samples and matrices can be very useful for researchers and may open the door to training artificial intelligence and prediction tools to assist in the optimization of sample preparation for targeted analysis.

## Supporting Information Available

Further information on chemicals and materials, additional experimental details, GC-IRMS validation results, and additional data on QCM dry mass sensing experiments (PDF)
