# Supplementary material for: Quartz Crystal Microbalance as a Holistic Detector for Quantifying Complex Organic Matrices during Liquid Chromatography: 2. Compound-Specific Isotope Analysis
Source: Anal Chem. 2024 May 3;96(19):7436–43. doi: 10.1021/acs.analchem.3c05441 (PMC11099894; doi:10.1021/acs.analchem.3c05441)
Supplement: Supplementary file 1 — ac3c05441_si_001.pdf [file ac3c05441_si_001.pdf]

Supporting Information for
Analytical Chemistry

Quartz Crystal Microbalance as a Holistic Detector for Quantifying Complex Organic Matrices during Liquid Chromatography: 2. Compound Specific Isotope Analysis

Christopher Wabnitz, Wei Chen, Martin Elsner, and Rani Bakkour*

Lichtenbergstr. 4, 85748 Garching, Germany. Technical University of Munich, Germany; TUM School of Natural Sciences, Department of Chemistry, Chair of Analytical Chemistry and Water Chemistry

*Corresponding author: rani.bakkour@tum.de
phone +49 89 289 54502, fax +49 89 2180 78255

26 Pages, 17 Figures, 17 Tables

Contents

S1 Chemicals, Materials, and Standard Solutions 2

S2 Extraction of Riverine NOM 3

S3 HPLC-QCM Dry Mass Sensing 3

S4 Compound Specific Isotope Analysis: Method Detection Limits, $\Delta\partial^{13}$C Data, and Chromatogram Background Data 5

S5 Details on HPLC Gradients and QCM Dry Mass Sensing Results 8

S1 Chemicals, Materials, and Standard Solutions

All chemicals and materials purchased commercially and used in this work are summarized in Table S1. Additional information on the selected model analytes can be found in Table S2.

Table S1 List of reagents, solvents, and analytical standards.

Chemical	Purity/Grade	Supplier
2,6-dichlorobenzamide (BAM)	PESTANAL®, anal. st.	Sigma-Aldrich
atrazine (ATZ)	PESTANAL®, anal. st.	Sigma-Aldrich
azoxystrobin (AZOX)	PESTANAL®, anal. st.	Sigma-Aldrich
boscalid (BOSC)	PESTANAL®, anal. st.	Sigma-Aldrich
caffeine (CAF), USGS63		USGS
desethylatrazine (DEA)	PESTANAL®, anal. st.	Sigma-Aldrich
desisopropylatrazine (DIA)	PESTANAL®, anal. st.	Sigma-Aldrich
methanol	≥99%	Sigma-Aldrich
simazine (SIM)	PESTANAL®, anal. st.	Sigma-Aldrich
sodium chloride	≥99.5%	Fisher Scientific

anal. st. = analytical standard

Ultrapure H_2O (18.2 MΩ cm at 25 °C) was obtained from a Milli-Q® direct reference H_2O purification system from Merck MilliPore (Burlington, USA). Stock solutions of analytical standards (1 g/L) were prepared in CH_3OH and stored at -18 °C.

Table S2 Additional information on the selected model analytes including the chemical formula, the isotope signature of the specific batch used, the molecular weight, the $\log K_{OW}$ and the charge at pH7.

Name	Formula	Isotope Signature	Molecular Weight [g/mol]	$\log K_{OW}$ [-]	Charge pH 7 [-]
BAM	$C_7H_5Cl_2NO$	n.a.	190.02	0.77	n
ATZ	$C_8H_{14}ClN_5$	-29,56	215.68	2.61	n
AZOX	$C_{22}H_{17}N_3O_5$	n.a.	403.4	2.50	n
BOSC	$C_{18}H_{12}Cl_2N_2O$	n.a.	343.2	2.96	n
CAF	$C_8H_{10}N_4O_2$	-1,17	194.19	-0.07	n
DEA	$C_6H_{10}ClN_5$	-29,39	187.63	1.51	n
DIA	$C_5H_8ClN_5$	-36,78	173.60	1.50	n
SIM	$C_7H_{12}ClN_5$	n.a.	201.66	2.18	n

n.a. = not available, n = neutral.

S2 Extraction of Riverine NOM

Surface water samples (Wiesäckerbach, latitude 48.269009, longitude 11.667976, Garching, Germany) were filtered using glass microfiber filter membranes (1.2 µm particle retention, 47 mm diameter, Whatman, UK). NOM was extracted using Oasis HLB SPE material (Waters, 200 mg, 6 cc) and an automated SPE system (Smart Prep Extractor, Horizon Technology, USA). The extraction was performed using conventional SPE conditions at 5 mL/min. The Oasis HLB cartridges were dried overnight under vacuum and eluted using 5 mL of CH_3OH. The eluates were combined, reduced under a gentle stream of nitrogen at 30 °C, and stored at -18 °C. TOC analysis (TOC-L, Shimadzu, Japan) was used to determine the carbon content of the eluate. Elemental analysis (EURO-EA, HEKATech, Germany) was used to determine the percentage of carbon in the extracted NOM (see Table S3).

Table S3 Elemental analysis of NOM extracted from Wiesäckerbach using Oasis HLB.

Element	Content in %
Carbon	51.20
Hydrogen	6.56
Nitrogen	2.02
Sulfur	0.51
Oxygen	39.71

S3 HPLC-QCM Dry Mass Sensing

A Nexera XR HPLC system (Shimadzu, Japan) was used for chromatographic separation. It consists of a solvent delivery module (LC-20AD, Shimadzu, Japan), a diode array detector (DAD) (SPD-M20A, Shimadzu, Japan), and a fraction collector (FRC-10A, Shimadzu, Japan). A microfluidic spray-dryer and a QCM were connected to the HPLC. The mobile phase was split after the DAD detector using a post-column adjustable flow splitter (ASI 610-PO10-01, 50:1 to 1000:1 Split Ratio, Analytical Scientific Instruments, USA). A fraction collector was connected to the high flow port, and a microfluidic spray-dryer to the low flow port. The microfluidic spray-dryer was fabricated in-house using a standard polydimethylsiloxane (PDMS) soft lithography approach.[1] Using the spray-dryer, the mobile phase was sprayed onto a 5 MHz QCM crystal (100RX1, Cr/Au, Stanford Research Systems, USA) placed in a frequency counter (QCM200, gate time: 0.1 s, Stanford Research Systems, USA). QCM dry mass sensing was performed as described previously in the companion paper.[1] In short, each measurement consisted of a blank run (200 µL of 25/75 CH_3OH/water (v/v)), the sample measurement, and a one-point calibration under the same chromatographic conditions with the eluents containing NaCl (c_{cal} = 300 mg/L) to continuously spray a constant mass concentration onto the QCM. For the data evaluation, a previously published Matlab script was used.[1] After correcting the sample (f_{sample}) and the calibration measurement (f_{cal}) using the blank measurement (f_{blank}), the 1st derivative ($\partial/\partial t$) was taken from the corrected frequencies Δf_{sample} and Δf_{cal}. Then, 1st derivatives

were smoothed using a Savitzky-Golay filter (polynomial order 3, 301 points). To get the mass concentration of the sample, the smoothed 1st derivative of the sample measurement was divided by the smoothed 1st derivative of the calibration measurement and multiplied by the concentration of the calibration solution (c_{cal}).

S4 Compound Specific Isotope Analysis: Method Detection Limits, $\Delta\partial^{13}C$ Data, and Chromatogram Background Data

Table S4 HPLC gradient used for purifying NOM sample prior to GC-C-IRMS measurement.

t/ min				
0	7.5	15	16.5	18
% of CH_3OH				
10	90	90	90	90

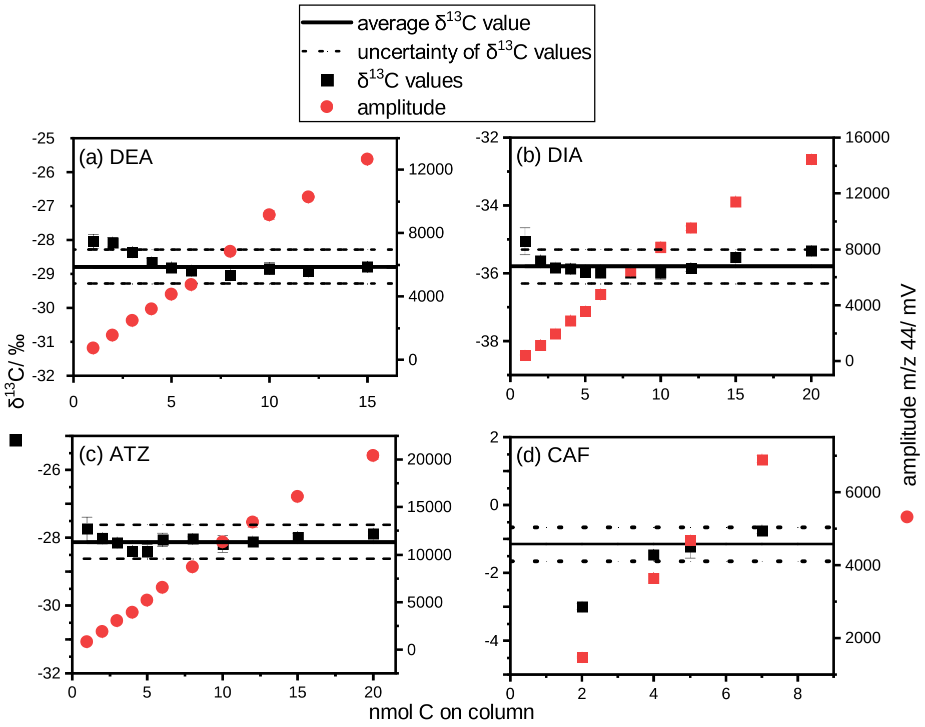

Figure S1 Determination of Method detection limits (nmol C) for GC-IRMS measurements with an uncertainty limit of ±0.5‰ (dotted lines) for four analytes: (a) DEA, (b) DIA, (c) ATZ, and (d) CAF. The measured $\partial^{13}C$ values are shown in black, and the respective peak amplitude in red (m/z 44 in mV). Isotope values and amplitudes are arithmetic means of at least triplicate measurements with their respective standard deviation.

Table S5 Method detection limits (nmol C) for GC-IRMS measurements determined according to the moving mean procedure with an uncertainty limit of ±0.5‰ and the corresponding analyte amplitude at m/z 44 in mV.

Analyte	MQL nmol C on column	Amplitude/ mV
ATZ	1	818 ± 24
DIA	2	1172 ± 156
DEA	3	2489 ± 78
CAF	4	3643 ± 71

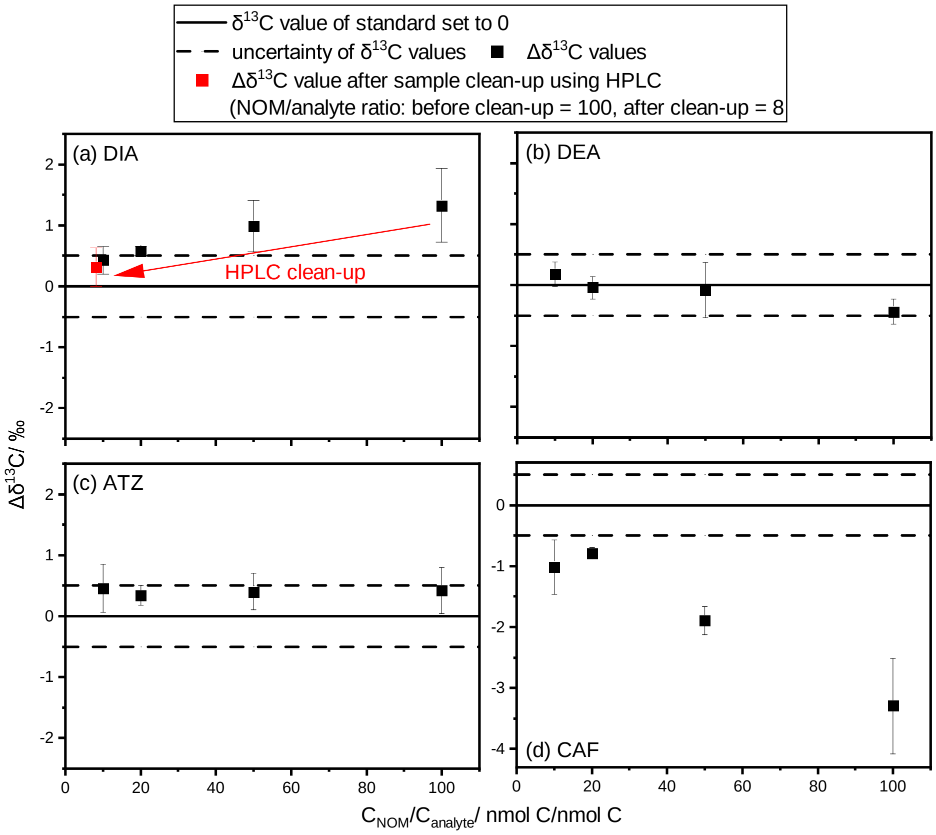

Figure S2 Deviations of carbon isotope values ($\Delta\partial^{13}$C in black) of (a) DIA, (b) DEA, (c) ATZ, and (d) CAF during the measurement of samples containing NOM in different amounts (NOM/analyte ratios: 10, 20, 50, 100) from the values of standard measurements. The dotted black lines show the uncertainty limit of carbon isotope analysis using GC-IRMS (±0.5‰). The error bars resemble the 95% confidence interval of triplicate measurements. The red data point shows a measurement after an HPLC clean-up using 10-60-90 gradient and column 1 of a sample containing NOM/analyte ratio 100 with an expected final NOM/analyte ratio of 8 after the clean-up.

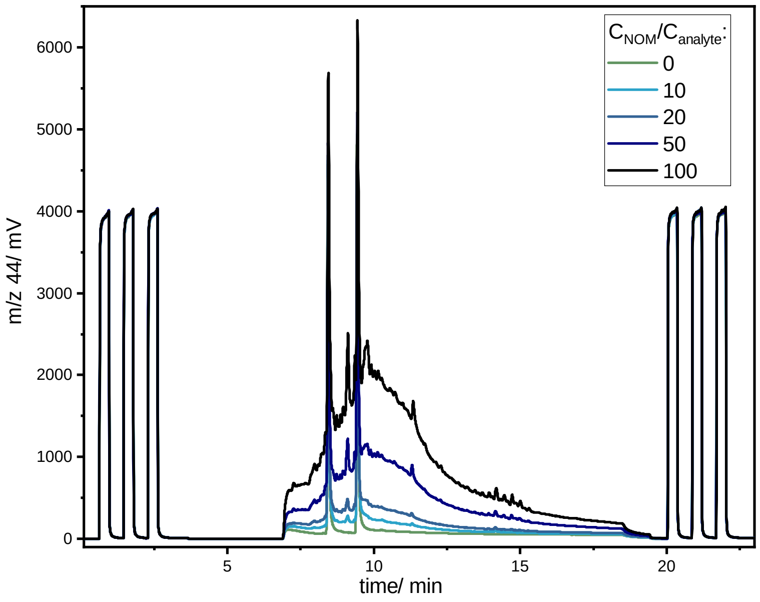

Figure S3 GC-IRMS chromatograms of DEA and CAF for different $C_{NOM}/C_{analyte}$ ratios.

S5 Details on HPLC Gradients and QCM Dry Mass Sensing Results

Table S6 22 HPLC gradients used to optimize separation of analytes and NOM on column 1. Mobile phase consists of CH_3OH and water.

t/ min				
0	7.5	15	16.5	18
% of CH_3OH				
10	30	60	90	90
10	30	70	90	90
10	30	80	90	90
10	30	90	90	90
10	40	60	90	90
10	40	70	90	90
10	40	80	90	90
10	40	90	90	90
10	50	60	90	90
10	50	70	90	90
10	50	80	90	90
10	50	90	90	90
10	60	60	90	90
10	60	70	90	90
10	60	80	90	90
10	60	90	90	90
10	70	70	90	90
10	70	80	90	90
10	70	90	90	90
10	80	80	90	90
10	80	90	90	90
10	90	90	90	90

Table S7 7 HPLC gradients used to optimize separation of analytes and NOM on column 2. Mobile phase consists of CH_3OH and water.

t/ min				
0	7.5	15	16.5	18
% of CH_3OH				
10	30	60	90	90
10	40	80	90	90
10	50	60	90	90
10	60	70	90	90
10	70	90	90	90
10	80	90	90	90
10	90	90	90	90

Table S8 Theoretical plate number for gradient 10-70-90.

	CAF	BAM	DIA	DEA	SIM	ATZ	AZOX	BOSC
Column 1	822	800	2061	5639	11294	13795	28775	27699
Column 2	10384	10605	15990	28947	41653	49415	50835	54224

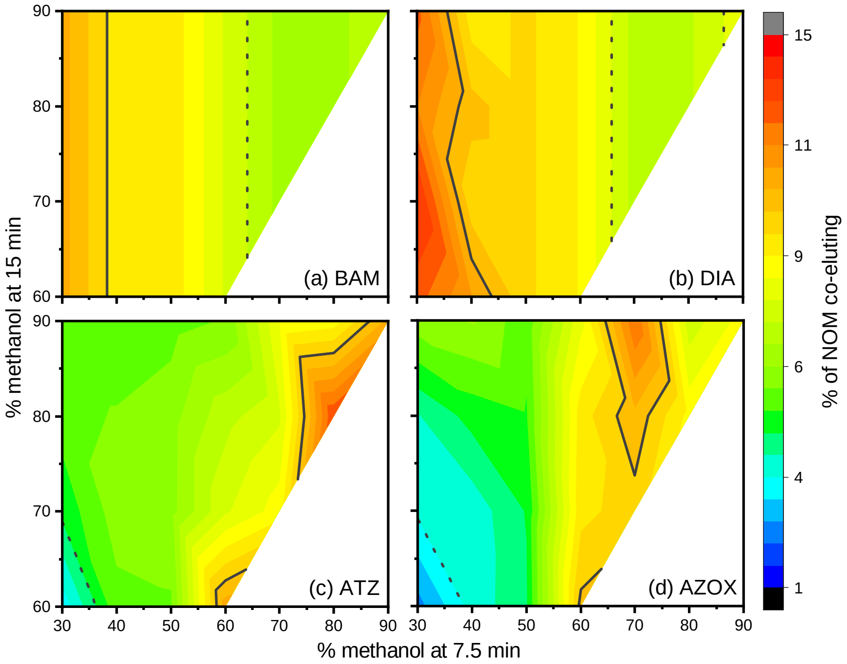

Figure S4 The NOM co-elution in % is plotted for the 22 different gradients for 4 analytes ((a): BAM, (b): DIA, (c): ATZ, (d): AZOX). The x-axis represents the % of methanol at minute 7.5 during the HPLC clean-up and the y-axis % of methanol at minute 15.

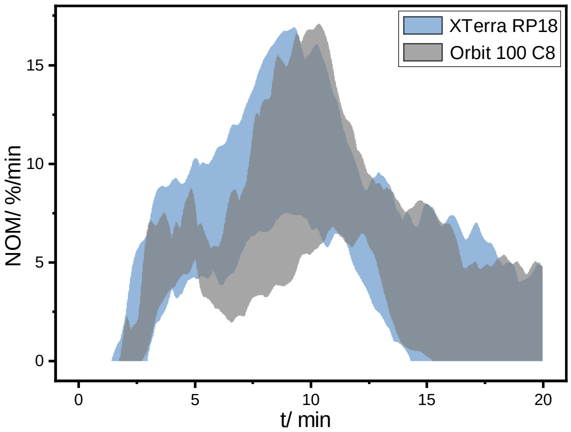

Figure S5 Comparison of NOM retention on column 1 (blue) and column 2 (grey). The bands resemble all measured NOM values (in %/min) between the maximal and the minimal value determined for 22 gradients in case of column 1 (XTerra RP18 Column, 150×3.0 mm, 3.5 µm, pore size 125 Å) and 7 gradients in case of column 2 (Orbit 100 C8 Column, 150×3.0 mm, 3.5 µm, pore size 100 Å).

Table S9 NOM co-elution in % shown for 22 HPLC gradients on XTerra RP18.

Gradient	CAF	BAM	DIA	DEA
10-30-60	14.75 ± 0.83	10.22 ± 0.80	11.72	10.62
10-30-70	14.75 ± 0.83	10.22 ± 0.80	12.63	9.79
10-30-80	14.75 ± 0.83	10.22 ± 0.80	11.02	8.90
10-30-90	14.75 ± 0.83	10.22 ± 0.80	11.67	8.59
10-40-60	12.34 ± 0.98	8.74 ± 0.67	10.54	10.24
10-40-70	12.34 ± 0.98	8.74 ± 0.67	9.17	9.58
10-40-80	12.34 ± 0.98	8.74 ± 0.67	9.69 ± 0.27	10.48 ± 0.90
10-40-90	12.34 ± 0.98	8.74 ± 0.67	8.67	9.29
10-50-60	11.25 ± 0.71	8.82 ± 0.72	9.13 ± 0.81	10.87 ± 0.50
10-50-70	11.25 ± 0.71	8.82 ± 0.72	9.13 ± 0.81	10.26
10-50-80	11.25 ± 0.71	8.82 ± 0.72	9.13 ± 0.81	10.22
10-50-90	11.25 ± 0.71	8.82 ± 0.72	9.13 ± 0.81	8.72
10-60-60	8.16 ± 0.14	7.45 ± 0.14	8.47 ± 1.06	11.02
10-60-70	8.16 ± 0.14	7.45 ± 0.14	8.47 ± 1.06	11.58
10-60-80	8.16 ± 0.14	7.45 ± 0.14	8.47 ± 1.06	10.36
10-60-90	8.16 ± 0.14	7.45 ± 0.14	8.47 ± 1.06	10.27
10-70-70	7.97 ± 0.80	6.35 ± 0.58	6.81 ± 0.78	9.66
10-70-80	7.97 ± 0.80	6.35 ± 0.58	6.81 ± 0.78	9.17
10-70-90	7.97 ± 0.80	6.35 ± 0.58	6.81 ± 0.78	8.56
10-80-80	7.96 ± 0.48	6.38 ± 0.46	6.92 ± 0.63	8.72 ± 0.85
10-80-90	7.96 ± 0.48	6.38 ± 0.46	6.92 ± 0.63	8.72 ± 0.85
10-90-90	8.58	6.81	7.82	7.20

Table S10 NOM co-elution in % shown for 22 HPLC gradients on XTerra RP18.

Gradient	SIM	ATZ	AZOX	BOSC
10-30-60	6.57	3.18	2.30	3.24
10-30-70	6.47	4.65	3.61	3.42
10-30-80	7.41	5.24	3.97	2.82
10-30-90	5.32	5.04	5.80	4.61
10-40-60	8.01	5.31	3.69	1.97
10-40-70	7.64	5.76	3.68	2.56
10-40-80	6.47 ± 0.55	5.56 ± 0.62	4.70 ± 0.28	3.82 ± 0.26
10-40-90	6.23	5.02	6.05	5.67
10-50-60	7.23 ± 0.70	5.43 ± 0.42	4.41 ± 0.81	2.36 ± 0.42
10-50-70	8.09	5.82	4.54	2.00
10-50-80	8.13	5.81	4.96	2.66
10-50-90	7.83	5.28	5.15	3.32
10-60-60	11.59	10.29	9.64	6.10
10-60-70	9.26	7.42	8.83	4.25
10-60-80	7.92	6.82	8.75	5.85
10-60-90	7.29	5.55	7.86	5.84
10-70-70	9.95	8.25	9.28	6.91
10-70-80	8.53	7.41	9.87	7.35
10-70-90	8.40	8.00	11.50	8.68
10-80-80	10.89	11.97	8.33	7.73
10-80-90	9.59 ± 0.43	8.24 ± 0.54	7.20 ± 0.61	6.63 ± 0.69
10-90-90	8.34	10.15	8.24	7.55

Table S11 NOM co-elution in % shown for 7 HPLC gradients on Orbit 100 C8.

Gradient	CAF	BAM	DIA	DEA
10-30-60	7.43	7.53	8.79	6.93
10-40-80	5.04	6.02	5.72	7.15
10-50-60	4.50	6.33	7.20	13.23
10-60-70	4.62	6.01	6.54	7.70
10-70-90	4.69	5.48	5.28	7.57
10-80-90	3.16	4.59	5.13	7.01
10-90-90	4.91	4.86	4.74	7.92

Table S12 NOM co-elution in % shown for 7 HPLC gradients on Orbit 100 C8.

Gradient	SIM	ATZ	AZOX	BOSC
10-30-60	3.57	2.92	2.65	1.94
10-40-80	6.33	5.05	3.50	2.70
10-50-60	8.90	2.30	0.60	0.18
10-60-70	9.95	6.84	2.47	1.72
10-70-90	8.01	5.97	10.40	6.83
10-80-90	8.05	6.87	8.30	6.55
10-90-90	9.02	8.17	7.61	5.85

Table S13 Minimal NOM co-elution dependent on the number of gradients screened (from 3 to 22 gradients). All gradient mixes include convex, concave, and linear gradients. The number of gradients that is sufficient to find the optimal conditions within a precision of 1% is marked bold.

Number of Gradients	CAF	BAM	DIA	DEA
3	**8.2**	**6.8**	7.8	**7.2**
5	8.0	6.4	**6.8**	7.2
7	8.0	6.4	6.8	7.2
10	8.0	6.4	6.8	7.2
12	8.0	6.4	6.8	7.2
15	8.0	6.4	6.8	7.2
18	8.0	6.4	6.8	7.2
22	8.0	6.4	6.8	7.2

Table S14 Minimal NOM co-elution dependent on the number of gradients screened (from 3 to 22 gradients). All gradient mixes include convex, concave, and linear gradients. The number of gradients that is sufficient to find the optimal conditions within a precision of 1% is marked bold.

Number of Gradients	SIM	ATZ	AZOX	BOSC
3	6.6	**3.2**	**2.3**	3.2
5	**6.2**	3.2	2.3	**2.0**
7	6.2	3.2	2.3	2.0
10	5.3	3.2	2.3	2.4
12	5.3	3.2	2.3	2.4
15	5.3	3.2	2.3	2.0
18	5.3	3.2	2.3	2.0
22	5.3	3.2	2.3	2.0

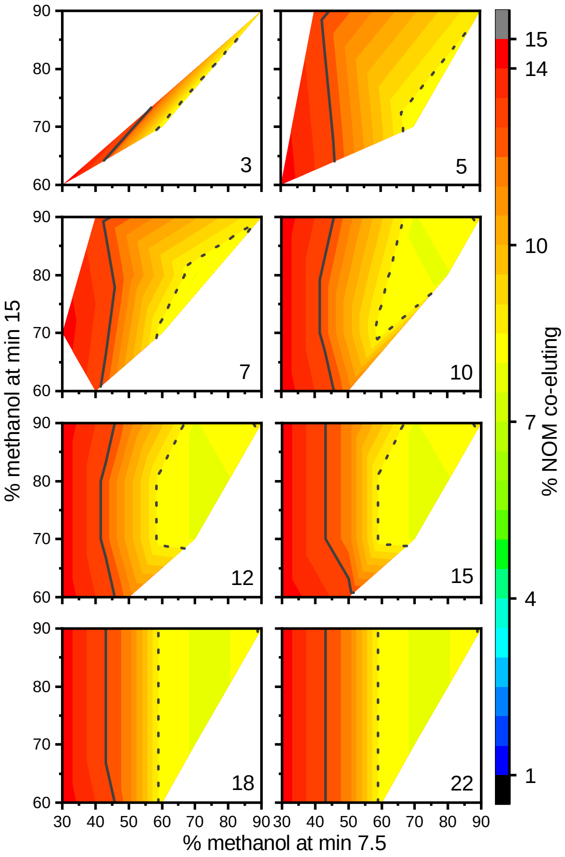

Figure S6 Gradient screening of CAF using 3, 5, 7, 10, 12, 15, 18, or 22 different gradients.

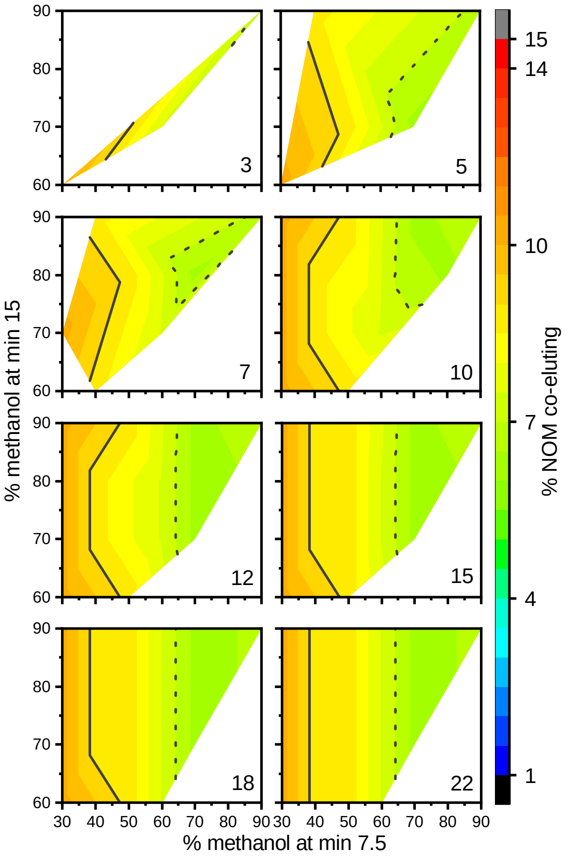

Figure S7 Gradient screening of BAM using 3, 5, 7, 10, 12, 15, 18, or 22 different gradients.

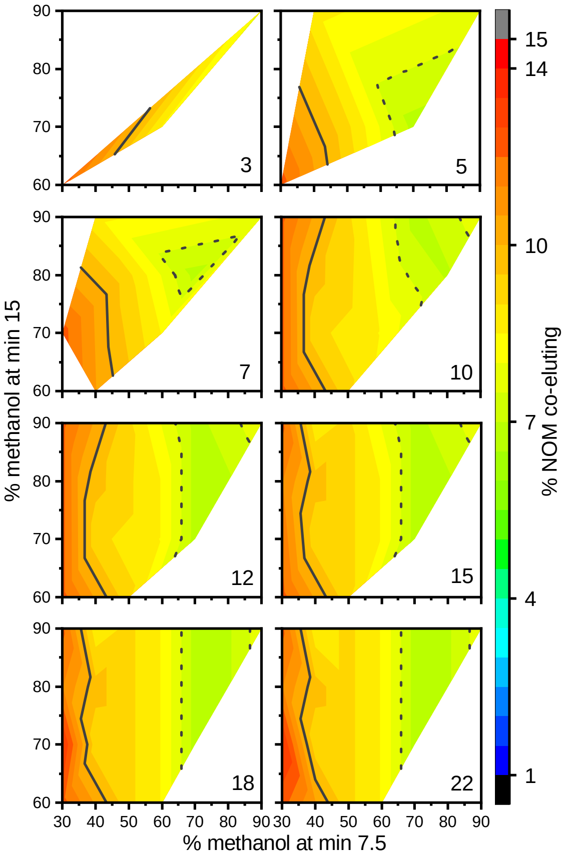

Figure S8 Gradient screening of DIA using 3, 5, 7, 10, 12, 15, 18, or 22 different gradients.

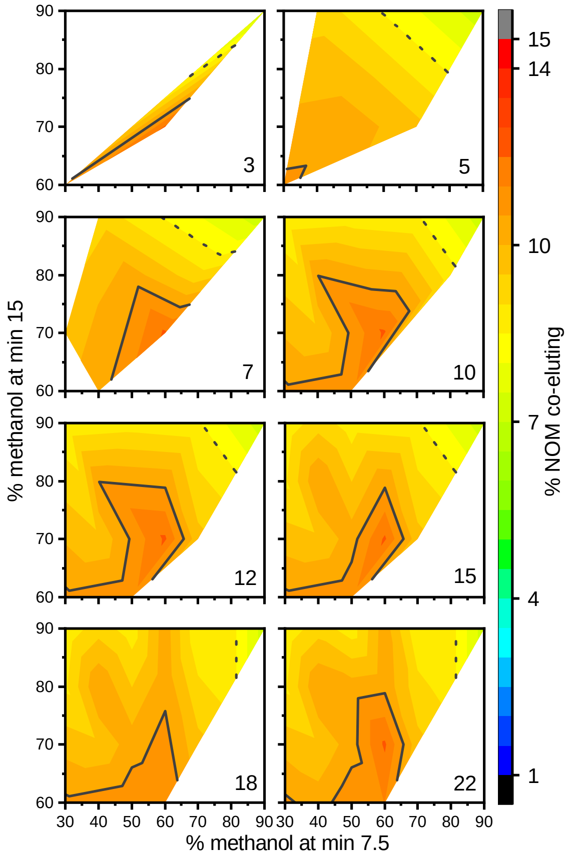

Figure S9 Gradient screening of DEA using 3, 5, 7, 10, 12, 15, 18, or 22 different gradients.

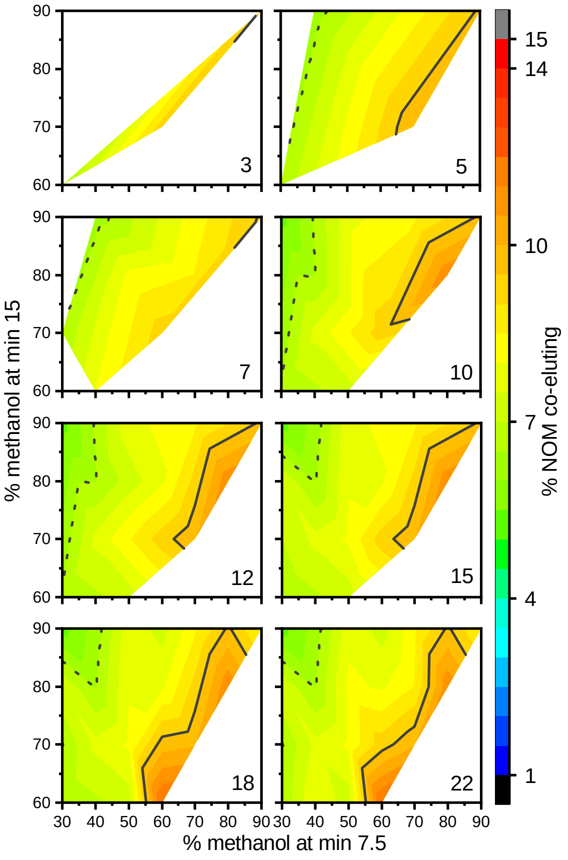

Figure S10 Gradient screening of SIM using 3, 5, 7, 10, 12, 15, 18, or 22 different gradients.

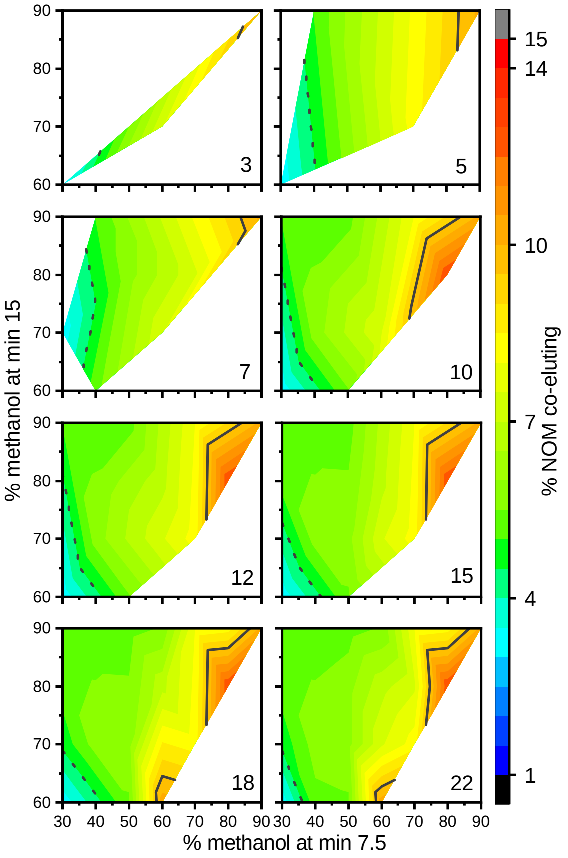

Figure S11 Gradient screening of ATZ using 3, 5, 7, 10, 12, 15, 18, or 22 different gradients.

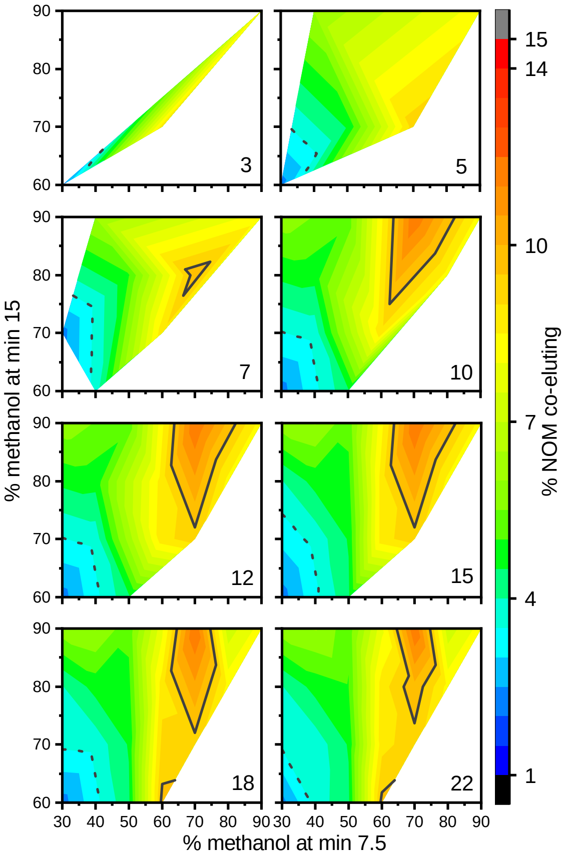

Figure S12 Gradient screening of AZOX using 3, 5, 7, 10, 12, 15, 18, or 22 different gradients.

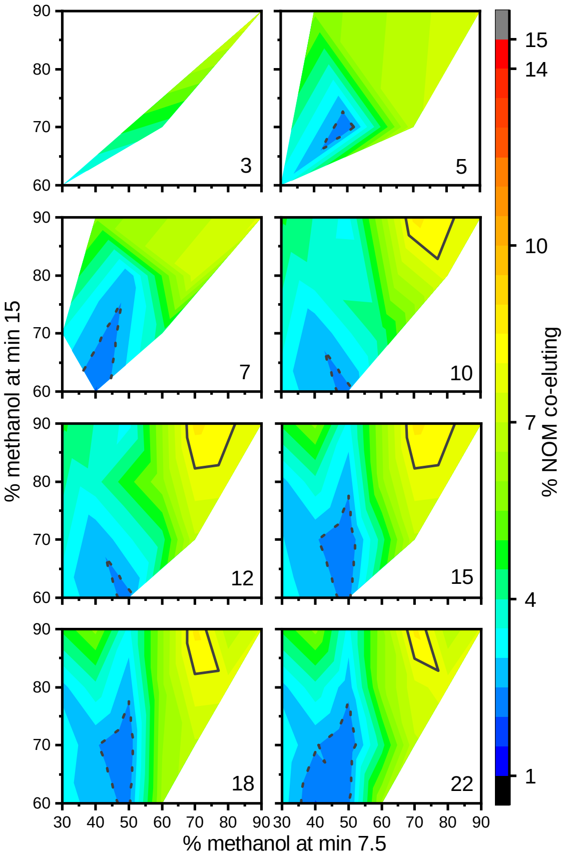

Figure S13 Gradient screening of BOSC using 3, 5, 7, 10, 12, 15, 18, or 22 different gradients.

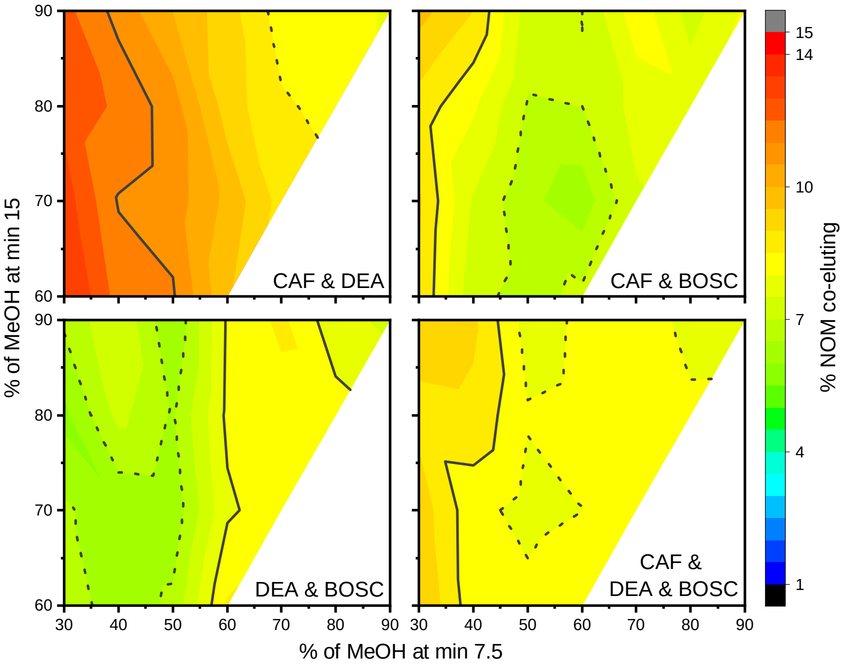

Figure S14 NOM co-elution average for HPLC purification of two or three compounds (early eluting: CAF, middle eluting: DEA, late eluting: BOSC).

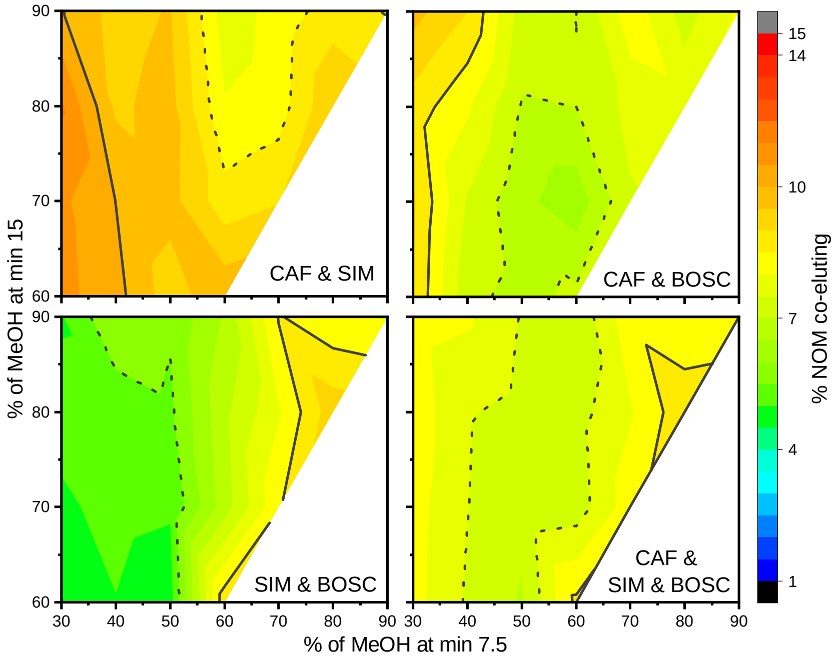

Figure S15 NOM co-elution average for HPLC purification of multiple compounds (early eluting: CAF, middle eluting: SIM, late eluting: BOSC).

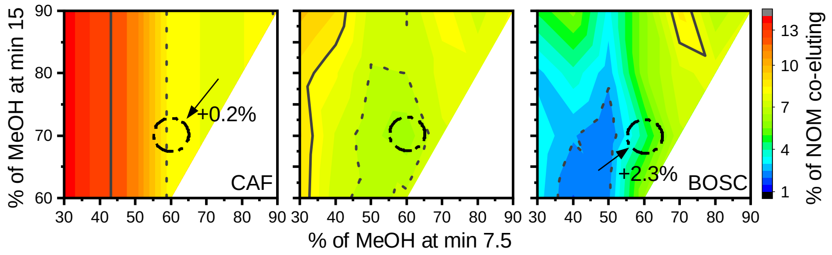

Figure S16 Gradient screening for individual compound (CAF, left panel; BOSC, right panel) and simultaneous purification of multiple compounds (CAF+BOSC, middle panel). The dashed circles show the minimum NOM co-elution for simultaneous optimization of both compounds, whereas the arrows show the increase in NOM co-elution when moving from individual to multiple.

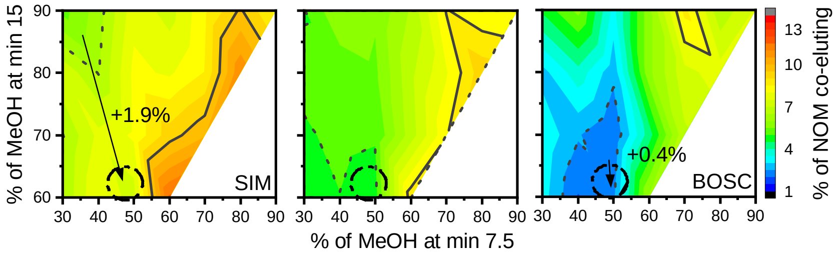

Figure S17 Gradient screening for individual compound (SIM, left panel; BOSC, right panel) and simultaneous purification of multiple compounds (SIM+BOSC, middle panel). The dashed circles show the minimum NOM co-elution for simultaneous optimization of both compounds, whereas the arrows show the increase in NOM co-elution when moving from individual to multiple.

Table S15 Higher NOM co-extraction during an optimized multi compound HPLC purification on XTerra RP18 in comparison to the optimized HPLC purification for a single compound is shown as optimization loss in % NOM (loss/ %). The different compounds are classified into early, middle, and late eluting.

compound			loss/%		
early	middle	late	early	middle	late
CAF	DEA		0.6	0	
CAF	SIM		0.2	2.0	
BAM	DEA		0.5	0	
BAM	SIM		1.1	2.0	
CAF		ATZ	0.2		2.4
CAF		AZOX	0		4.9
CAF		BOSC	0.2		2.3
BAM		ATZ	1.1		2.4
BAM		AZOX	2.4		1.4
BAM		BOSC	2.4		0
	DEA	ATZ		1.4	1.9
	DEA	AZOX		1.7	1.7
	DEA	BOSC		1.7	0.9
	SIM	ATZ		1.3	0
	SIM	AZOX		1.3	0
	SIM	BOSC		1.9	0.4
BAM	DEA	ATZ	0	1.4	4.8
BAM	DEA	ATZ	2.4	2.1	1.8
BAM	DEA	ATZ	2.5	1.5	2.1
BAM	DEA	AZOX	2.4	2.4	1.4
BAM	DEA	AZOX	2.5	1.5	2.9
BAM	DEA	AZOX	0.5	0	5.9
BAM	SIM	ATZ	3.9	1.3	0
BAM	SIM	ATZ	2.4	0.9	1.8
BAM	SIM	ATZ	1.1	2.0	2.4
BAM	SIM	AZOX	3.9	1.3	0
BAM	SIM	AZOX	2.4	1.2	2.4
CAF	DEA	BOSC	3.3	1.5	1.4
CAF	DEA	BOSC	0	1.5	4.7
CAF	DEA	BOSC	3.3	3.1	0
CAF	SIM	BOSC	3.3	1.9	0.4
CAF	SIM	BOSC	0.2	2.0	3.9

Table S16 Higher NOM co-extraction during an optimized multi compound HPLC purification on Orbit 100 C8 in comparison to the optimized HPLC purification for a single compound is shown as optimization loss in % NOM (loss/ %). The different compounds are classified into early, middle, and late eluting.

compound			loss/%		
early	middle	late	early	middle	late
CAF	DEA		0	0.1	
CAF	SIM		4.3	0	
BAM	DEA		0	0.1	
BAM	SIM		2.9	0	
CAF		ATZ	1.3		0
CAF		AZOX	1.3		0
CAF		BOSC	1.3		0
BAM		ATZ	1.7		0
BAM		AZOX	1.7		0
BAM		BOSC	1.7		0
	DEA	ATZ		0	0.6
	DEA	AZOX		0	2.1
	DEA	BOSC		0	1.8
	SIM	ATZ		0	0.6
	SIM	AZOX		0	2.1
	SIM	BOSC		0	1.8
BAM	DEA	ATZ	2.9	0	0.6
BAM	DEA	AZOX	2.9	0	2.1
BAM	DEA	AZOX	1.4	0.8	1.9
BAM	SIM	ATZ	2.9	0	0.6
BAM	SIM	AZOX	2.9	0	2.1
CAF	DEA	BOSC	1.5	0.8	1.5
CAF	SIM	BOSC	4.3	0	1.8

Table S17 Overview of columns used in the field of CSIA during HPLC purification of compounds present in different sample matrices.

publication	compounds	matrix	column
Schreglmann et al.[2]	pesticides	water	ODS (30) Ultracarb
Piper et al.[3]	steroids	urine	LiChrospher 100 RP18
Melsbach et al.[4]	pesticides	water	Synergi Hydro-RP
Tripp et al.[5]	amino acids	bones	Waters Symmetry or Supelco Discovery HS F5
Lalonde et al.[6]	steroids	urine	Zorbax Eclipse XDB Phenyl column and Zorbax Eclipse XDB-C18 columns
Swalethorp et al.[7]	amino acids	fish	Primesep A
Yun et al.[8]	pesticides	plants	Zorbax Eclips XDB C18
Cheng et al.[9]	hexabromocyclododecane	no	Zorbax SB-C18
Shrivastava et al.[10]	steroids	urine	LiChroCART
Akamatsu et al.[11]	glucose	sake	VG50-4E
Murata et al.[12]	aroma compounds	green tea	CAPCELLPAK ODS
Zhu et al.[13]	decabrominated diphenyl ether	no	Zorbax Eclipse Plus-C18
Iannella et al.[14]	prednisolone or prednisone	pharmaceutical formulation	ACE® C18
Saccon et al.[15]	phenols	air	Supelco Supelcosil™ LC-18
Broek and McCarthy[16]	amino acids	proteinaceous samples	SiELC Primesep A
Drenzek et al.[17]	PCBs	bacteria culture	Chrompack Chromsep Omnipher 5 C18

References

[1] Wabnitz, C.; Chen, W.; Canavan, A.; Bakkour, R. Quartz Crystal Microbalance as Holistic Detector for Quantifying Complex Organic Matrices During Liquid Chromatography: 1. Coupling, Characterization, and Validation. *Anal. Chem.* **2023**, *under revision.*

[2] Schreglmann, K.; Hoeche, M.; Steinbeiss, S.; Reinnicke, S.; Elsner, M. Carbon and nitrogen isotope analysis of atrazine and desethylatrazine at sub-microgram per liter concentrations in groundwater. *Anal. Bioanal. Chem.* **2013**, *405*, 2857–2867.

[3] Piper, T.; Mareck, U.; Geyer, H.; Flenker, U.; Thevis, M.; Platen, P.; Schanzer, W. Determination of 13C/12C ratios of endogenous urinary steroids: method validation, reference population and application to doping control purposes. *Rapid Commun. Mass Spectrom.* **2008**, *22 14*, 2161–75.

[4] Melsbach, A.; Pittois, D.; Bayerle, M.; Daubmeier, M.; Meyer, A. H.; Hölzer, K.; Gallé, T.; Elsner, M. Isotope fractionation of micropollutants during large-volume extraction: heads-up from a critical method evaluation for atrazine, desethylatrazine and 2,6-dichlorobenzamide at low ng/L concentrations in groundwater. *Isot. Environ. Health Stud.* **2020**, *57*, 35 – 52.

[5] Tripp, J. A.; McCullagh, J. S. O.; Hedges, R. E. M. Preparative separation of underivatized amino acids for compound-specific stable isotope analysis and radiocarbon dating of hydrolyzed bone collagen. *J. Sep. Sci.* **2006**, *29 1*, 41–8.

[6] Lalonde, K.; Barber, A.; Ayotte, C. Two-dimensional HPLC purification of underivatized urinary testosterone and metabolites for compound specific stable carbon isotope analysis. *Drug Test. Anal.* **2020**,

[7] Swalethorp, R.; Aluwihare, L. I.; Thompson, A. R.; Ohman, M. D.; Landry, M. R. Errors associated with compound specific δ15N analysis of amino acids in preserved fish samples purified by high pressure liquid chromatography. *bioRxiv* **2019**,

[8] Yun, H. Y.; Won, E.-J.; Choi, J.; Cho, Y.; Lim, D. J.; Kim, I.-S.; Shin, K. Stable Isotope Analysis of Residual Pesticides via High Performance Liquid Chromatography and Elemental Analyzer–Isotope Ratio Mass Spectrometry. *Molecules* **2022**, *27*.

[9] Cheng, G.; Gao, S.; Gao, Y.; Yu, Z.; Peng, P. Compound-specific stable carbon isotope analysis of hexabromocyclododecane diastereoisomers using gas chromatography-isotope ratio mass spectrometry. *Rapid Commun. Mass Spectrom.* **2019**,

[10] Shrivastava, A.; Jain, S.; lal Sahu, P.; Shukla, S.; Sahu, K. Improved Method for Differentiation of Synthetic and Natural Endogenous Anabolic Steroids using Gas Chromatography Isotope Ratio Mass Spectrometry (GC/C/IRMS) followed by Two-Fold High Performance Liquid Chromatography (HPLC) Cleanup Method: A Perspective. *Indian J. Pharm. Educ. Res.* **2019**,

[11] Akamatsu, F.; Igi, Y.; Fujita, A. Separation and Purification of Glucose in Sake for Carbon Stable Isotope Analysis. *Food Analytical Methods* **2020**, *13*, 885–891.

[12] Murata, A.; Engelhardt, U. H.; Fleischmann, P.; Yamada, K.; Yoshida, N.; Juchelka, D.; Hilkert, A.; Ohnishi, T.; Watanabe, N.; Winterhalter, P. Purification and gas chromatography-combustion-isotope ratio mass spectrometry of aroma compounds from green tea products and comparison to bulk analysis. *J. Agric. Food Chem.* **2013**, *61 47*, 11321–5.

[13] Zhu, X.; Zhong, Y.; Wang, H.; Li, D.; Deng, Y.; Gao, S.; Peng, P. Compound-specific carbon isotope analysis for mechanistic characterization of debromination of decabrominated diphenyl ether. *Rapid Commun. Mass Spectrom.* **2020**,

[14] Iannella, L.; Botré, F.; Colamonici, C.; Curcio, D.; Ciccarelli, C.; Mazzarino, M.; de la Torre, X. Carbon isotopic characterization of prednisolone and prednisone pharmaceutical formulations: implications in antidoping analysis. *Drug Test. Anal.* **2020**,

[15] Saccon, M.; Busca, R.; Facca, C.; Huang, L.; Irei, S.; Kornilova, A.; Lane, D. A.; Rudolph, J. Method for the determination of concentration and stable carbon isotope ratios of atmospheric phenols. *Atmos. Meas. Tech.* **2013**, *6*, 2965–2974.

[16] Broek, T. A. B.; McCarthy, M. D. A new approach to δ15N compound-specific amino acid trophic position measurements: preparative high pressure liquid chromatography technique for purifying underivatized amino acids for stable isotope analysis. *Limnol. Oceanogr.: Methods* **2014**, *12*.

[17] Drenzek, N. J.; Eglinton, T. I.; Wirsen, C. O.; Sturchio, N. C.; Heraty, L. J.; Sowers, K. R.; Wu, Q.; May, H. D.; Reddy, C. M. Invariant chlorine isotopic signatures during microbial PCB reductive dechlorination. *Environ Pollut.* **2004**, *128*, 445–448.
